# Association between aspartate aminotransferase to alanine aminotransferase ratio and 28-day mortality of ICU patients: A retrospective cohort study from MIMIC-IV database

**DOI:** 10.1371/journal.pone.0324904

**Published:** 2025-05-23

**Authors:** Yanping Wang, Yan Xu

**Affiliations:** Department of Pharmacy, The Affiliated People’s Hospital of Ningbo University, Ningbo, Zhejiang, China; Universitas Muhammadiyah Aceh, INDONESIA

## Abstract

**Background:**

Prior studies have linked the aspartate aminotransferase to alanine aminotransferase ratio (AAR) with negative health outcomes in the elderly and specific populations. However, the impact of AAR on the prognosis of the entire population in the intensive care unit (ICU) remains unclear. This study aimed to determine the correlation between AAR and the mortality among adult ICU patients.

**Method:**

Patient data were retrieved from the Medical Information Mart for Intensive Care IV (MIMIC-IV) database and stratified into quartiles by AAR. Survival analysis using the Kaplan-Meier curves was conducted to compare survival across quartiles. The primary outcome was 28-day mortality, with secondary outcomes including 60-day, 90-day, and 365-day mortality, along with ICU-free, ventilator-free, and vasopressor-free days within the first 28 days. The association between AAR and mortality was evaluated using Cox proportional hazards regression analysis complemented by a restricted cubic spline. Furthermore, the eICU Collaborative Research Database (eICU-CRD) was used as an external validation cohort for sensitivity analysis.

**Result:**

The study included 20,225 patients with a mean age of 63.7 ± 17.5 years. Kaplan-Meier analysis indicated a higher risk of 28-day mortality for patients with higher AAR (log-rank P < 0.001). After adjusting for confounders, the AAR was significantly related to 28-day mortality (HR = 1.04, 95% CI: 1.03–1.06, P < 0.001) and other mortality benchmarks, exhibiting an inverted L-shaped relationship. The inflection point of the AAR for 28-day mortality was 2.60. Below this threshold, each unit increase in the AAR was associated with a 19% rise in the risk of 28-day mortality (HR = 1.19, 95% CI: 1.11–1.27, P < 0.001), with a plateau observed above this threshold. Subgroup and sensitivity analyses further confirmed the robustness and generalizability of the study.

**Conclusion:**

AAR demonstrated a significant association with 28-day, 60-day, 90-day, and 365-day mortality, characterized by an inverted L-shaped pattern.

## Introduction

Alanine aminotransferase (ALT) and aspartate aminotransferase (AST) are common indicators used in liver function tests. ALT is specifically located in the cytoplasm of hepatocytes, while AST is found in both the cytoplasm and mitochondria [1]. Initially introduced to diagnose acute viral hepatitis, the AST/ALT ratio (AAR) has become a key biomarker for liver diseases and is significantly linked to poor prognoses in affected individuals [2–6]. In contrast to ALT, which is predominantly found in the liver, AST is abundantly present in a variety of tissues such as the liver, heart, muscle, kidney, and brain [1]. Recently, more studies have begun to explore the relationship between AAR and nonliver diseases. High AAR has been linked to an elevated risk of sarcopenia [7]. Another study demonstrated that a high AAR is correlated with increased mortality among elderly populations, exhibiting stronger predictive power for mortality compared to individual AST or ALT levels [8]. Furthermore, elevated AAR has been associated with increased mortality in patients with hypertension, diabetes, sepsis, coronavirus disease of 2019 (COVID-19), heart failure, acute myocardial infarction, coronary artery disease, major burn, traumatic brain injuries, acute ischemic stroke, peripheral arterial occlusive disease, and cancers [9–21].

ICU patients, at high risk of mortality, often have comorbidities, severe trauma, systemic inflammation, frailty, malnutrition, and sarcopenia [22]. Previous studies have associated elevated AAR with poor outcomes across multiple diseases, but these studies have primarily focused on specific critical conditions or elderly patients [11,23–25]. However, the relationship between AAR and the prognosis of the entire adult population in the ICU remains unclear. This study aims to explore the association between AAR and mortality in adult ICU population, offering a more detailed evaluation of its prognostic potential.

## Methods

### Study design

We performed a retrospective cohort analysis, utilizing data sourced from the MIMIC-IV database (version 2.2), eliminating the need for the approval of the ethics committee or informed consent of the patient. This comprehensive, high-quality database, developed and sustained by the Massachusetts Institute of Technology, covers deidentified health data from ICU patients at Beth Israel Deaconess Medical Center (BIDMC) from 2008 to 2019. Ethical approval, an informed consent waiver, and research resource sharing approval were granted by the BIDMC Institutional Review Board [26 27]. Access to the database was provided to one of our researchers after completion of the training on protection of human research participants (record ID: 59403724). Our research adhered to the Strengthening the Reporting of Observational studies in Epidemiology (STROBE) statement guidelines for observational studies.

### Study population

**Fig 1:**
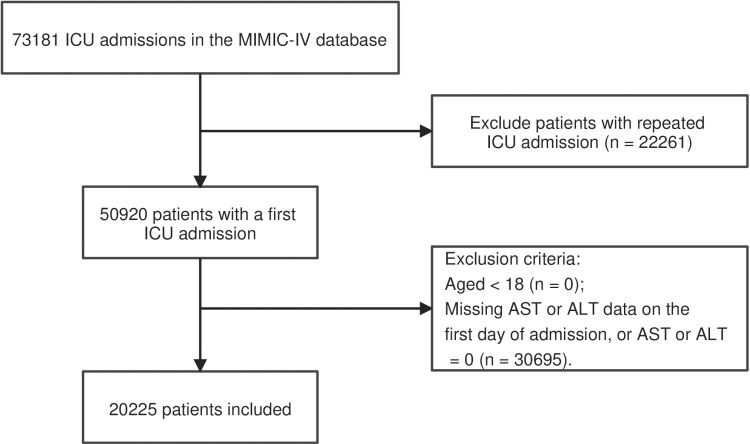
Flowchart of the patient selection.

The MIMIC-IV database yielded 73,181 ICU admissions, from which we selected the first instance for patients with recurrent stays, resulting in 50,920 included patients after exclusions. Patients with AST or ALT levels of 0, or missing AST and ALT values on admission day, were excluded. Our final cohort comprised 20,225 patients aged 18 and older, divided into quartile-based groups according to their AAR on the day of ICU admission. The flowchart for patient screening is presented in Fig 1.

### Variable extraction

We sourced data from the MIMIC-IV database, focusing on the initial 24-hour period following ICU admission. This included demographics such as gender, age, weight, race, and marital status, along with lifestyle factors like smoking status. Vital signs recorded were heart rate, systolic blood pressure (SBP), diastolic blood pressure (DBP), respiratory rate, and SpO2. Laboratory tests encompassed glucose, hemoglobin, platelets, white blood cell (WBC), anion gap, blood urea nitrogen (BUN), creatinine, electrolytes, prothrombin time (PT), AST, and ALT. Clinical scores like the Charlson comorbidity index, Oxford acute severity of illness score (OASIS), and Sequential organ failure assessment (SOFA) were also included. AAR was calculated as AST divided by ALT, with comorbidities identified through the International Classification of Diseases, Ninth Revision (ICD-9) and ICD-10 codes. Sepsis diagnosis adhered to the ‘Third International Consensus Definition for Sepsis and Septic Shock’. Furthermore, we extracted information on hospital and ICU stay lengths and patient outcomes, including mortality rates and days without ICU, ventilator, and vasopressor support up to 28 days. All variables included in the analysis had less than 5% missing values.

### Primary outcome and secondary outcomes

The primary outcome was 28-day mortality. Secondary outcomes were 60-day, 90-day, and 365-day mortality, as well as ICU-free, ventilator-free, and vasopressor-free days within the first 28 days.

### Sensitivity analysis

To enhance the generalizability of the study, we incorporated an external validation cohort using the eICU-CRD (version 2.0). This multi-center ICU database comprises data from 200,859 ICU admissions across 208 hospitals in the United States between 2014 and 2015. The eICU-CRD is de-identified to ensure patient privacy and has been approved by the Institutional Review Board of the Massachusetts Institute of Technology, with a waiver of informed consent granted [28,29]. Notably, the eICU-CRD follows patients only up to discharge; therefore, 28-day mortality data are unavailable. As a result, we used in-hospital mortality as the outcome for our study. Of the 200,859 ICU admissions in the database, 61,105 patients were included in the final analysis (see S1 Fig). Data extracted from the eICU-CRD focused on the first 24 hours after ICU admission, including demographics, vital signs, laboratory data, comorbidities, and severity of illness.

### Statistical analysis

The baseline characteristics of the participants were delineated based on the quartiles of the AAR. For continuous variables that exhibited a normal distribution, the mean and standard deviation were reported; for those that did not, the median and interquartile range (IQR) were used. Categorical variables were presented in absolute numbers with percentages. Statistical analyses, such as One-way ANOVA, Kruskal-Wallis H test, Chi-square test, or Fisher’s exact test, were utilized to compare variations in baseline characteristics among the groups. Multiple imputation was used to replace missing values based on three replications. Additionally, sensitivity analyses were conducted by excluding missing data from the dataset.

Kaplan-Meier survival curves were constructed and compared across varying AAR groups using the log-rank test. Cox proportional hazard regression models were employed to investigate the association between AAR and mortality, with results expressed as hazard ratios (HR) and 95% confidence intervals (CI). Four models were utilized for result stability verification: Model 1 without covariate adjustments; Model 2 Adjusted for age, gender, weight, race, smoking, and marital status; Model 3 expanded on Model 2 by including heart rate, SBP, DBP, respiratory rate, SpO2, hemoglobin, platelets, WBC, anion gap, BUN, creatinine, potassium, sodium, and PT; Model 4 further adjusted for a broad spectrum of comorbidities and clinical scores. The inclusion of covariates was driven by clinical relevance, significance in univariate analysis (S1 Table), and their potential to alter the effect estimate by more than 10%. Multicollinearity was detected using the variance inflation factor (VIF), where a VIF of 5 or higher signaled multicollinearity. Furthermore, a restricted cubic spline (RCS) regression model with four knots was applied to examine the non-linear relationship between AAR and mortality, and a threshold effect analysis was conducted to identify the inflection point. Subgroup analyses were also performed using stratified Cox regression, with interaction assessed via a likelihood ratio test.

Our statistical procedures were executed with R (Version 4.2.1, http://www.R-project.org, The R Foundation) alongside the Free Statistics software (Version 1.9.1, http://www.clinicalscientists.cn/freestatistics). Differences with two-sided P < 0.05 were considered statistically significant.

## Results

### Baseline characteristics

Our study included 20,225 participants with a mean age of 63.7 ± 17.5 years. Of these, 56.8% were male and 63.5% were white. The median values for AST and ALT were recorded at 45.0 IU/L (25.0, 115.0) and 31.0 IU/L (17.0, 74.0), respectively. By categorizing the participants based on quartiles of AAR at ICU admission (Q1: < 1.035, Q2: 1.035–1.441, Q3: 1.441–2.055, Q4 ≥ 2.056), a detailed analysis of the baseline characteristics of the patients is presented in Table 1. The AAR levels for the four groups were 0.8 (0.6, 0.9), 1.2 (1.1, 1.3), 1.7 (1.6, 1.9), 2.7 (2.3, 3.5), respectively. Compared to the lower group, the highest AAR group exhibited lower body weight, a lower proportion of individuals married, and higher levels of heart rate, respiratory rate, WBC, anion gap, BUN, creatinine, PT, OASIS, and SOFA. They also had lower levels of SBP, DBP, SpO2, glucose, hemoglobin, and platelets. Furthermore, this group had a higher prevalence of myocardial infarction, congestive heart failure, coronary heart disease, sepsis, liver disease, and renal disease, as well as longer hospital and ICU stays. The 28-day, 60-day, 90-day, and 360-day mortality all increased from the Q1 to the Q4 group. In addition, ICU-free, ventilator-free, and vasopressor-free days up to 28 days all decreased from the Q1 to the Q4 group.

**Table 1:** Baseline characteristics of patients grouped according to AAR quartiles.

Characteristics	Total (n = 20255)	AAR quartiles				P value
		Q1 < 1.035 (n = 5064)	Q2 (1.035–1.441) (n = 5063)	Q3 (1.441–2.055) (n = 5061)	Q4 ≥ 2.056 (n = 5067)	
**Demographics**						
Male, n (%)	11495 (56.8)	3126 (61.7)	2810 (55.5)	2731 (54)	2828 (55.8)	< 0.001
Age, years	63.7 ± 17.5	61.0 ± 17.6	64.3 ± 17.6	65.3 ± 17.8	64.2 ± 16.7	< 0.001
Weight, Kg	82.0 ± 26.4	85.4 ± 32.4	81.8 ± 25.0	80.0 ± 23.1	80.7 ± 23.7	< 0.001
Race, n (%)						0.009
Non-white	7391 (36.5)	1795 (35.4)	1794 (35.4)	1866 (36.9)	1936 (38.2)	
White	12864 (63.5)	3269 (64.6)	3269 (64.6)	3195 (63.1)	3131 (61.8)	
Marital status, n (%)						< 0.001
Married	8339 (41.2)	2208 (43.6)	2134 (42.1)	2010 (39.7)	1987 (39.2)	
Others	11916 (58.8)	2856 (56.4)	2929 (57.9)	3051 (60.3)	3080 (60.8)	
Smoking, n (%)	3515 (17.4)	858 (16.9)	892 (17.6)	924 (18.3)	841 (16.6)	0.125
**Vital signs**						
Heart rate, bpm	86.9 ± 17.1	86.5 ± 17.4	85.9 ± 17.3	86.7 ± 16.8	88.6 ± 16.9	< 0.001
SBP, mmHg	118.1 ± 17.4	120.1 ± 17.1	119.6 ± 17.4	117.9 ± 17.6	114.7 ± 16.9	< 0.001
DPB, mmHg	64.9 ± 11.8	67.0 ± 11.7	65.4 ± 11.7	64.1 ± 11.8	63.0 ± 11.7	< 0.001
Respiratory rate, bpm	19.8 ± 4.1	19.7 ± 3.9	19.6 ± 4.0	19.7 ± 4.0	20.1 ± 4.3	< 0.001
SpO2, %	91.2 ± 7.5	91.6 ± 5.9	91.6 ± 6.3	91.0 ± 8.1	90.6 ± 9.1	< 0.001
**Laboratory data**						
Glucose, mg/dL	128.1 (106.5, 162.0)	128.0 (107.9, 166.2)	130.0 (106.5, 163.8)	128.0 (106.0, 161.3)	126.9 (105.7, 157.2)	< 0.001
Hemoglobin, g/dL	10.2 ± 2.4	10.8 ± 2.4	10.5 ± 2.3	10.0 ± 2.3	9.6 ± 2.3	< 0.001
Platelets, K/µL	170.0 (111.0, 233.0)	189.0 (135.0, 251.0)	179.0 (123.0, 240.0)	164.0 (109.0, 229.0)	143.0 (83.0, 208.0)	< 0.001
WBC, K/µL	12.3 (8.6, 17.5)	12.0 (8.5, 16.8)	11.9 (8.4, 16.8)	12.5 (8.6, 17.7)	12.8 (8.8, 18.6)	< 0.001
Anion gap, mmol/L	17.5 ± 5.5	16.7 ± 4.8	17.1 ± 5.3	17.5 ± 5.5	18.5 ± 6.2	< 0.001
BUN, mg/dL	22.0 (14.0, 37.0)	21.0 (14.0, 35.0)	21.0 (14.0, 35.0)	23.0 (15.0, 38.0)	25.0 (15.0, 41.0)	< 0.001
Creatinine, mg/dL	1.1 (0.8, 1.8)	1.0 (0.8, 1.6)	1.1 (0.8, 1.6)	1.1 (0.8, 1.8)	1.3 (0.9, 2.2)	< 0.001
Calcium, mg/dL	8.6 ± 1.0	8.6 ± 0.9	8.6 ± 0.9	8.6 ± 1.0	8.6 ± 1.0	< 0.001
Potassium, mmol/L	4.6 ± 0.9	4.5 ± 0.8	4.6 ± 0.9	4.6 ± 0.9	4.7 ± 1.0	< 0.001
Chloride, mmol/L	105.6 ± 6.6	105.5 ± 6.4	105.8 ± 6.4	105.8 ± 6.6	105.2 ± 7.1	< 0.001
Sodium, mmol/L	139.9 ± 5.3	140.1 ± 5.1	140.1 ± 5.1	139.8 ± 5.3	139.4 ± 5.7	< 0.001
PT, s	14.5 (12.7, 18.4)	14.0 (12.4, 16.6)	14.1 (12.4, 17.3)	14.7 (12.7, 18.6)	15.7 (13.2, 20.9)	< 0.001
ALT, IU/L	31.0 (17.0, 74.0)	50.0 (28.0, 123.0)	28.0 (17.0, 64.0)	24.0 (15.0, 54.0)	26.0 (14.0, 59.0)	< 0.001
AST, IU/L	45.0 (25.0, 115.0)	38.0 (22.0, 87.0)	35.0 (22.0, 81.0)	42.0 (25.0, 99.0)	78.0 (39.0, 198.0)	< 0.001
AST/ALT ratio	1.4 (1.0, 2.1)	0.8 (0.6, 0.9)	1.2 (1.1, 1.3)	1.7 (1.6, 1.9)	2.7 (2.3, 3.5)	< 0.001
**Comorbidities, n (%)**						
Myocardial infarct	3428 (16.9)	671 (13.3)	736 (14.5)	851 (16.8)	1170 (23.1)	< 0.001
Congestive heart failure	5393 (26.6)	1296 (25.6)	1283 (25.3)	1366 (27)	1448 (28.6)	< 0.001
Coronary heart disease	2182 (10.8)	440 (8.7)	515 (10.2)	548 (10.8)	679 (13.4)	< 0.001
Atrial fibrillation	3828 (18.9)	888 (17.5)	990 (19.6)	987 (19.5)	963 (19)	0.032
Peripheral vascular disease	2018 (10.0)	416 (8.2)	494 (9.8)	556 (11)	552 (10.9)	< 0.001
Cerebrovascular disease	2964 (14.6)	722 (14.3)	861 (17)	740 (14.6)	641 (12.7)	< 0.001
Dementia	768 (3.8)	183 (3.6)	204 (4)	197 (3.9)	184 (3.6)	0.632
Chronic pulmonary disease	4797 (23.7)	1258 (24.8)	1178 (23.3)	1219 (24.1)	1142 (22.5)	0.039
Rheumatic disease	662 (3.3)	147 (2.9)	137 (2.7)	200 (4)	178 (3.5)	0.001
Liver disease	4197 (20.7)	698 (13.8)	820 (16.2)	1087 (21.5)	1592 (31.4)	< 0.001
Renal disease	3938 (19.4)	868 (17.1)	967 (19.1)	1046 (20.7)	1057 (20.9)	< 0.001
Hypertension	12151 (60.0)	2983 (58.9)	3105 (61.3)	3089 (61)	2974 (58.7)	0.007
Diabetes	5734 (28.3)	1506 (29.7)	1499 (29.6)	1410 (27.9)	1319 (26)	< 0.001
Sepsis	11332 (55.9)	2535 (50.1)	2671 (52.8)	2895 (57.2)	3231 (63.8)	< 0.001
**Severity of illness**						
Charlson comorbidity index	6.0 (3.0, 8.0)	5.0 (3.0, 7.0)	5.0 (3.0, 7.0)	6.0 (4.0, 8.0)	6.0 (4.0, 8.0)	< 0.001
OASIS	33.0 ± 9.7	30.7 ± 9.1	32.4 ± 9.3	33.8 ± 9.7	35.2 ± 10.1	< 0.001
SOFA	4.0 (2.0, 7.0)	4.0 (2.0, 6.0)	4.0 (2.0, 7.0)	5.0 (2.0, 8.0)	6.0 (3.0, 9.0)	< 0.001
**Length of stay (LOS)**						
LOS in hospital, days	7.4 (4.1, 13.6)	6.8 (3.9, 12.4)	7.0 (3.9, 12.7)	7.7 (4.2, 13.9)	8.2 (4.6, 15.6)	< 0.001
LOS in ICU, days	2.2 (1.2, 4.5)	2.0 (1.1, 3.9)	2.1 (1.1, 4.1)	2.2 (1.2, 4.7)	2.6 (1.4, 5.3)	< 0.001
**Outcomes**						
28-day mortality, n (%)	3889 (19.2)	631 (12.5)	776 (15.3)	1078 (21.3)	1404 (27.7)	< 0.001
60-day mortality, n (%)	4675 (23.1)	792 (15.6)	974 (19.2)	1285 (25.4)	1624 (32.1)	< 0.001
90-day mortality, n (%)	5114 (25.2)	898 (17.7)	1086 (21.4)	1387 (27.4)	1743 (34.4)	< 0.001
360-day mortality, n (%)	6691 (33.0)	1277 (25.2)	1469 (29.0)	1788 (35.3)	2157 (42.6)	< 0.001
ICU-free days up to 28 days	25.2 (16.9, 26.6)	25.8 (22.4, 26.8)	25.5 (20.9, 26.7)	25.0 (13.2, 26.5)	23.8 (0.0, 26.2)	< 0.001
Ventilator-free days up to 28 days	28.0 (23.9, 28.0)	28.0 (27.0, 28.0)	28.0 (26.1, 28.0)	27.8 (21.5, 28.0)	27.3 (0.0, 28.0)	< 0.001
Vasopressor-free days up to 28 days	28.0 (25.0, 28.0)	28.0 (27.4, 28.0)	28.0 (26.7, 28.0)	28.0 (23.4, 28.0)	27.8 (0.0, 28.0)	< 0.001

Notes: Continuous variables are presented as mean ± SD if normally distributed, and median (IQR) if not normally distributed. Categorical variables are presented as n (%). Abbreviations: Q, quartile; bpm, beats per minute; SBP, systolic blood pressure; DBP, diastolic blood pressure; WBC, white blood cell; BUN, blood urea nitrogen; PT, prothrombin time; AST, aspartate aminotransferase; ALT, alanine aminotransferase; SOFA, sequential organ failure assessment; OASIS, Oxford acute severity of illness score.

### Survival analysis

**Fig 2:**
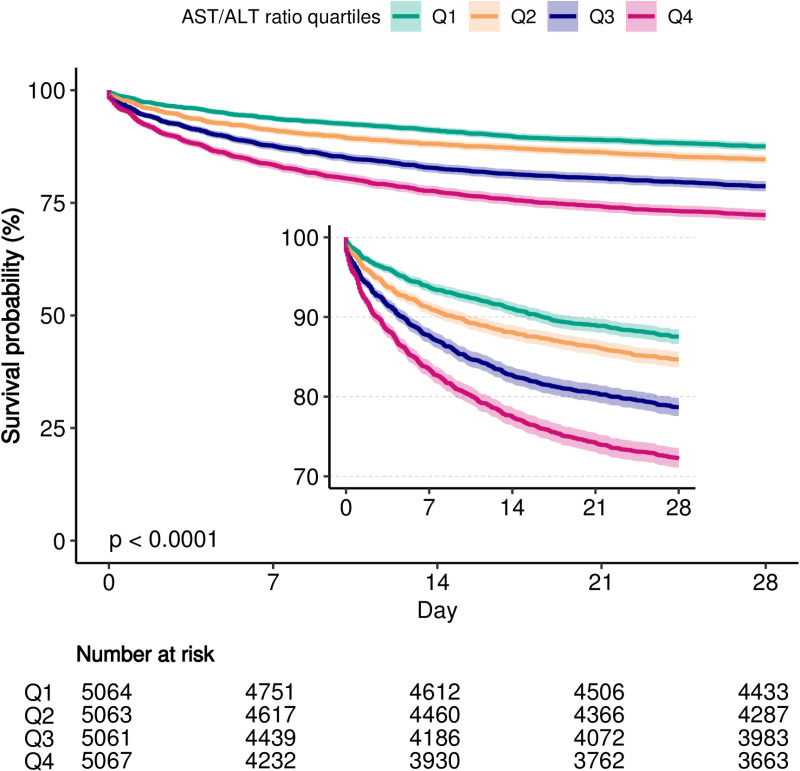
Kaplan-Meier survival analysis curves of 28-day mortality by AAR.

The Kaplan-Meier survival curves presented in Fig 2 reveal the link between the AAR quartiles and 28-day mortality. The mortality rates showed a statistically significant gradual increase from the Q1 to the Q4 groups (Q1: 12.5% vs. Q2: 15.3% vs. Q3: 21.3% vs. Q4: 27.7%, P < 0.0001). It is significant to note that patients with a higher AAR are at an elevated risk of mortality within the first 28 days.

### AAR and clinical outcomes

To ascertain the independent impact of AAR on 28-day mortality, four Cox proportional hazard models were utilized, as detailed in Table 2. In the unadjusted model 1, AAR exhibited a positive association with 28-day mortality (HR = 1.1, 95% CI: 1.09–1.10, P < 0.001). This link persisted in the subsequent adjusted models: Model 2 (HR = 1.1, 95% CI: 1.09–1.11), Model 3 (HR = 1.07, 95% CI: 1.06–1.09), and Model 4 (HR = 1.04, 95% CI: 1.03–1.06), each with a P-value below 0.001.

**Table 2:** Cox model analysis of AAR and 28-day mortality.

Variable	Model 1		Model 2		Model 3		Model 4	
	HR(95%)	P value	HR(95%)	P value	HR(95%)	P value	HR(95%)	P value
AAR	1.1 (1.09 ~ 1.10)	<0.001	1.1 (1.09 ~ 1.11)	<0.001	1.07 (1.06 ~ 1.09)	<0.001	1.04 (1.03 ~ 1.06)	<0.001
AAR quartiles								
Q1 < 1.035	1 (Reference)		1 (Reference)		1 (Reference)		1 (Reference)	
Q2 (1.035–1.441)	1.26 (1.13 ~ 1.40)	<0.001	1.16 (1.05 ~ 1.29)	0.005	1.13 (1.02 ~ 1.26)	0.02	1.08 (0.97 ~ 1.20)	0.158
Q3 (1.441–2.055)	1.81 (1.64 ~ 2.00)	<0.001	1.63 (1.48 ~ 1.80)	<0.001	1.43 (1.29 ~ 1.58)	<0.001	1.29 (1.17 ~ 1.43)	<0.001
Q4 ≥ 2.056	2.46 (2.24 ~ 2.70)	<0.001	2.28 (2.07 ~ 2.50)	<0.001	1.71 (1.55 ~ 1.88)	<0.001	1.41 (1.28 ~ 1.56)	<0.001

Model 1: no covariates were adjusted.

Model 2: adjusted for age, gender, weight, race, smoking, and marital status.

Model 3: adjusted for mode 2 + heart rate, SBP, DBP, Respiratory rate, SpO2, hemoglobin, platelets, WBC, aniongap, bun, creatinine, potassium, sodium, and PT.

Model 4: adjusted for mode 3 + myocardial infarct, congestive heart failure, atrial fibrillation, peripheral vascular disease, cerebrovascular disease, dementia, chronic pulmonary disease, liver disease, renal disease, hypertension, diabetes, sepsis, Charlson comorbidity index, OASIS, and SOFA.

For sensitivity analysis, AAR was categorized into quartiles, using Q1 serving as the reference group. In Model 4, the risk of 28-day mortality increased by 8%, 29%, and 41% for patients in Q2, Q3, and Q4, respectively (HR = 1.08, 95% CI: 0.97–1.20, P = 0.158; HR = 1.29, 95% CI: 1.17–1.43, P < 0.001; HR = 1.41, 95% CI: 1.28–1.56, P < 0.001). Additionally, the results remained consistent after the exclusion of 2447 patients, who constituted 12.1% of the total sample and had missing data, as detailed in the S2 Table.

Table 3 presents secondary outcomes, indicating that an elevated AAR was linked to increased 60-day, 90-day, and 365-day mortality, and reduced days free from ICU, ventilator, and vasopressor support within the first 28 days.

**Table 3:** Association between AAR and secondary outcome.

Variable	Coefficient (95%CI)				
	AAR continuous	AAR quartiles			
		Q1 < 1.035	Q2 (1.035–1.441)	Q3 (1.441–2.055)	Q4 ≥ 2.056
**60-day mortality**					
Crude	1.1 (1.09 ~ 1.11)	1 (Reference)	1.26 (1.15 ~ 1.39)	1.74 (1.59 ~ 1.9)	2.31 (2.12 ~ 2.51)
Adjusted^a^	1.05 (1.03 ~ 1.06)	1 (Reference)	1.09 (1 ~ 1.2)	1.25 (1.14 ~ 1.37)	1.37 (1.26 ~ 1.5)
**90-day mortality**					
Crude	1.1 (1.09 ~ 1.1)	1 (Reference)	1.24 (1.14 ~ 1.36)	1.66 (1.53 ~ 1.81)	2.2 (2.03 ~ 2.39)
Adjusted^a^	1.04 (1.03 ~ 1.06)	1 (Reference)	1.08 (0.99 ~ 1.19)	1.21 (1.11 ~ 1.31)	1.34 (1.23 ~ 1.45)
**365-day mortality**					
Crude	1.09 (1.09 ~ 1.1)	1 (Reference)	1.19 (1.1 ~ 1.28)	1.53 (1.43 ~ 1.65)	1.97 (1.84 ~ 2.11)
Adjusted^a^	1.04 (1.03 ~ 1.06)	1 (Reference)	1.06 (0.98 ~ 1.14)	1.14 (1.06 ~ 1.23)	1.27 (1.18 ~ 1.37)
**Vasopressor-free days up to 28 days**					
Crude	-1.28 (-1.4 ~ -1.16)	0 (Reference)	-0.81 (-1.23 ~ -0.39)	-2.57 (-2.99 ~ -2.14)	-4.65 (-5.07 ~ -4.23)
Adjusted^a^	-0.37 (-0.48 ~ -0.27)	0 (Reference)	0.1 (-0.26 ~ 0.46)	-0.52 (-0.89 ~ -0.16)	-1.21 (-1.59 ~ -0.83)
**ICU-free days up to 28 days**					
Crude	-1.24 (-1.35 ~ -1.13)	0 (Reference)	-0.81 (-1.21 ~ -0.41)	-2.55 (-2.95 ~ -2.14)	-4.55 (-4.95 ~ -4.15)
Adjusted^a^	-0.36 (-0.45 ~ -0.26)	0 (Reference)	0.09 (-0.25 ~ 0.42)	-0.57 (-0.9 ~ -0.23)	-1.2 (-1.55 ~ -0.85)
**Ventilator-free days up to 28 days**					
Crude	-1.25 (-1.37 ~ -1.14)	0 (Reference)	-0.85 (-1.27 ~ -0.43)	-2.64 (-3.06 ~ -2.22)	-4.58 (-5 ~ -4.16)
Adjusted^a^	-0.37 (-0.47 ~ -0.27)	0 (Reference)	0.04 (-0.32 ~ 0.4)	-0.64 (-1.01 ~ -0.28)	-1.24 (-1.62 ~ -0.86)

^a^Adjusted for all factors in Model 4 (age, gender, weight, race, smoking, marital status, heart rate, SBP, DBP, Respiratory rate, SpO2, hemoglobin, platelets, WBC, aniongap, bun, creatinine, potassium, sodium, PT, myocardial infarct, congestive heart failure, atrial fibrillation, peripheral vascular disease, cerebrovascular disease, dementia, Chronic pulmonary disease, liver disease, renal disease, hypertension, diabetes, sepsis, Charlson comorbidity index, OASIS, and SOFA).

### The analysis of inverted L-shaped association

**Fig 3:**
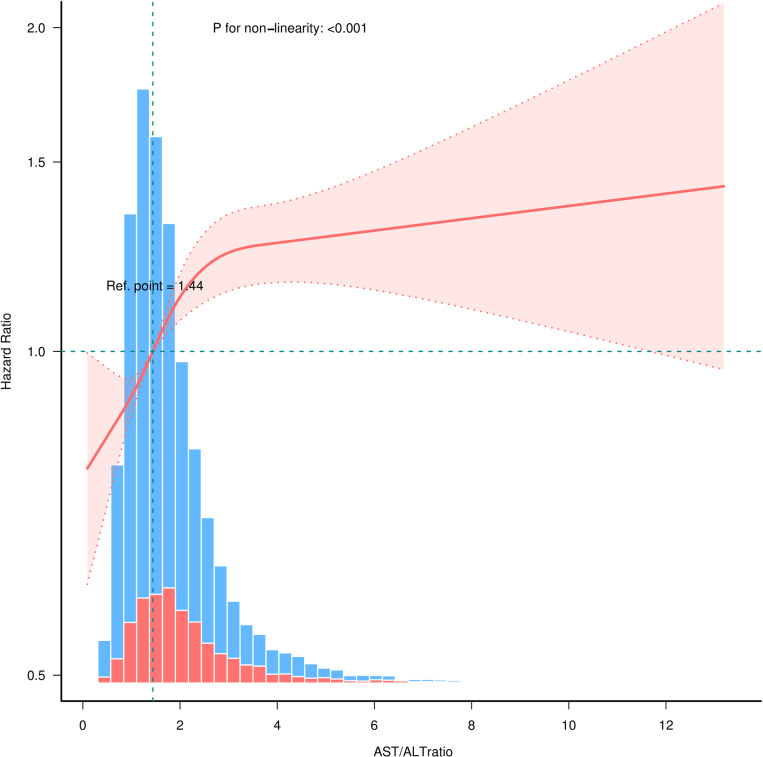
Restricted cubic spline regression analysis of AAR and 28-day mortality. Only 99.9% of the data is shown. The median AAR was defined as the reference standard. The pink area represents the 95% CI. Adjusted for all factors in Model 4.

Multivariable-adjusted RCS analyses suggested an inverted L-shaped association between AAR and 28-day mortality (P for non-linearity < 0.0001, Fig 3). This trend was also observed for 60-day, 90-day, and 365-day mortality (S2 Fig). The inflection point was ascertained using a two-piecewise linear regression model, as shown in Table 4. For an AAR less than 2.60, 28-day mortality increased with higher AAR (HR = 1.19, 95% CI: 1.11–1.27, P < 0.001). Beyond the inflection point, the association became non-significant, with a horizontal curve (HR = 1.02, 95% CI: 0.97–1.06, P = 0.498).

**Table 4:** Threshold effect analysis of AAR and 28-day mortality.

AAR ratio	Adjusted Model	
	HR (95%CI)	P value
< 2.60	1.19 (1.11 ~ 1.27)	< 0.001
≥ 2.60	1.02 (0.97 ~ 1.06)	0.498
Likelihood Ratio test		< 0.001

### Subgroup analysis

**Fig 4:**
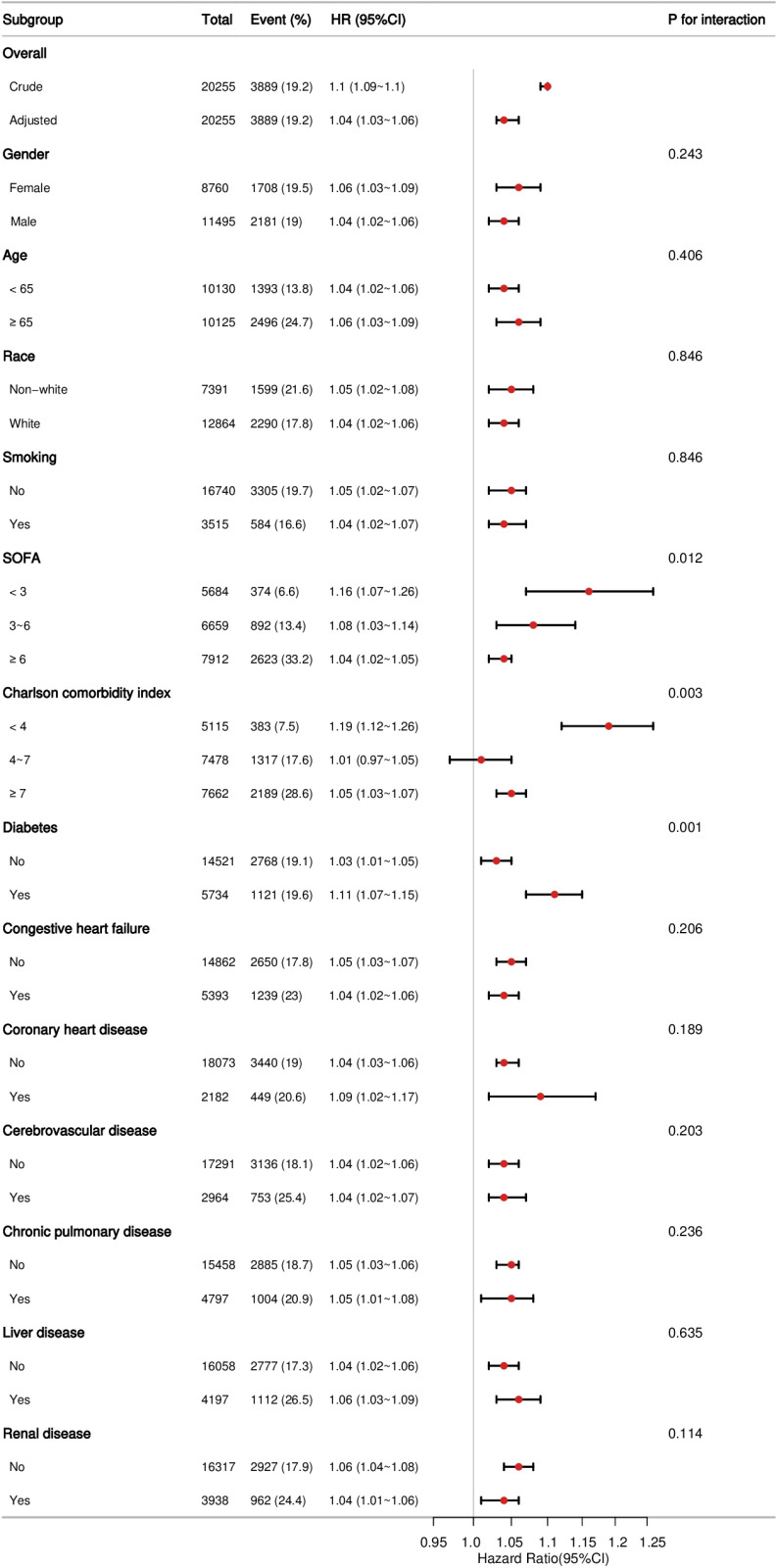
Forest plots of stratified analyses of AAR and 28-day mortality. Except for the stratification factor itself, each stratification factor was adjusted for all variables in Model 4.

To delve deeper into the association between the AAR and 28-day mortality, we analyzed various subgroups, including gender, age, race, smoking status, SOFA score, Charlson comorbidity index, and conditions such as heart failure, coronary heart disease, cerebrovascular disease, chronic pulmonary disease, liver disease, renal disease, and diabetes (see Fig 4). These analyses demonstrated that the relationship was robust and reliable in all subgroups. Significant interactions were observed in the SOFA score tertiles, the Charlson comorbidity index tertiles, and between patients with and without diabetes. Among diabetic patients and those with a lower Charlson comorbidity index and SOFA scores, the AAR exerts a notably greater impact on 28-day mortality.

### Sensitivity analysis

S3 Table provides a detailed analysis of the baseline characteristics of the included patients in the eICU-CRD. After adjusting for all confounders, the AAR was significantly associated with in-hospital mortality (HR = 1.04, 95% CI: 1.03–1.05, P < 0.001). Quartile analysis revealed a stepwise increase in mortality risk with higher AAR quartiles. Specifically, Q2 had an HR of 1.16 (95% CI: 1.06–1.27), Q3 showed an HR of 1.28 (95% CI: 1.18–1.39), and Q4 exhibited an HR of 1.51 (95% CI: 1.39–1.64) (see S4 Table). These findings demonstrate the increasing risk of in-hospital mortality associated with higher AAR quartiles. Multivariable-adjusted RCS analyses suggested an inverted L-shaped association between AAR and in-hospital mortality in the eICU-CRD (P for non-linearity < 0.0001, S3 Fig).

## Discussion

This retrospective cohort study confirmed that AAR is a significant independent predictor of mortality in ICU patients. A pronounced inverted L-shaped relationship was delineated between AAR and 28-day mortality, with an inflection point at 2.6. Mortality increased with AAR below this threshold and plateaued above it. The study’s subgroup and sensitivity analyses robustly affirm the correlation’s reliability and potency concerning the 28-day mortality. The research introduces a straightforward and effective biomarker that can be used for swift identification and intervention in patients at high risk.

A previous study based on the NHANES database showed that in elderly American community-based individuals (age ≥ 65 years), low levels of ALT and AST, along with high AAR, are associated with increased mortality, with AAR exhibiting the strongest predictive power for all-cause mortality [8]. Another study confirmed that a high AAR was an independent predictor of all-cause and cardiovascular mortality in a Japanese community-based population (age > 40 years), outperforming AST and ALT as standalone indicators [30]. A recent investigation into critically ill elderly Americans (age ≥ 65 years) uncovered a non-linear, saturation-based relationship between AAR and in-hospital mortality, with AAR levels below 1.80 correlating with increased mortality [25]. Our study, drawing from the MIMIC-IV database, includes a broader spectrum of critically ill patients, half of whom are under 65 years old. Analysis using RCS indicated an inverted L-shaped relationship between AAR and mortality at various time points (28-day, 60-day, 90-day, and 365-day) after adjusting for confounding factors. Elevated AAR levels, below the inflection point, were significantly and independently linked to higher mortality. Additionally, increased AAR levels were independently associated with an increased need for vasopressor support, ventilators, and prolonged ICU stays within 28 days. To our knowledge, this study is the first effort to definitively establish the AAR as a standalone mortality predictor in the adult ICU population.

Recent studies have also linked the elevated AAR to adverse outcomes in patients with various diseases. For instance, Maeda D. et al. reported a significant one-year mortality risk (HR = 1.57, 95% CI: 1.02–2.42) in the high AAR group compared to the low group in older Japanese heart failure patients [13]. Qin C. et al. found that in COVID-19 patients, an elevated AST/ALT ratio (≥1.38) at admission was linked to higher mortality risk and worse clinical status. Dynamic monitoring revealed that a sustained decrease in AAR during treatment suggests a favorable prognosis [31]. Another study linked higher AAR at stroke onset to increased 3-month and one-year mortality, as well as poor outcomes in patients with transient ischemic attack or acute ischemic stroke [18]. Zoppini G. et al. also discovered the AAR to be an independent risk factor for both all-cause and cardiovascular mortality in individuals with type 2 diabetes [10]. Our study extends these findings to critically ill ICU patients, conducting subgroup analyses based on the presence of conditions such as congestive heart failure, cerebrovascular disease, and diabetes. Notably, our subgroup analysis in patients with congestive heart failure (CHF) revealed results similar to those reported by Bian Y et al., who demonstrated that an elevated AAR within the first 24 h of ICU admission is independently associated with increased 28-day mortality in critically ill patients with CHF (HR = 1.18, 95% CI: 1.04–1.34) [24]. Furthermore,we found that an elevated AAR is an independent predictor of 28-day mortality, particularly pronounced in diabetic patients and those with lower Charlson comorbidity index and SOFA scores. The possible reasons for this outcome could be that patients with higher SOFA scores or Charlson comorbidity index exhibit a greater number of risk factors affecting mortality, thereby diminishing the independent effect of AAR on death. Further investigation is necessary to validate these findings and to elucidate the specific mechanisms underlying this relationship.

The mechanisms linking elevated AAR to increased mortality in ICU patients are not fully understood, but several hypotheses have been proposed. First, heightened AAR levels may signal mitochondrial dysfunction, potentially leading to elevated oxidative stress. AST, found in both the cytoplasmic and mitochondrial compartments of hypermetabolic tissues, and ALT, primarily in the hepatocyte cytoplasm, play crucial roles in carbohydrate and protein metabolism and participate in the auxiliary metabolic pathways of glycolysis [1]. If there is mitochondrial dysfunction, tissues rich in AST may substantially release AST, potentially leading to an elevated AAR. Our study observed a higher incidence of myocardial infarction, congestive heart failure, coronary artery disease, liver disease, and renal disease in patients with elevated AAR. An animal study reported that rats with high AAR had lower aerobic capacity and increased oxidative stress biomarker activity [32]. Additionally, mitochondrial dysfunction and oxidative stress can impair immune function, predispose to infections, and augment the release of inflammatory mediators. This has been confirmed in an animal study where mice with an elevated AAR exhibited mitochondrial damage, heightened oxidative stress, and increased expression of inflammatory cytokines IL-1β and IL-18 [33]. Furthermore, another study found a significant link between increased AAR and the levels of inflammatory cytokines such as IL-4, IL-6, and TNF-α in type 2 diabetes [34]. Our study also noted a higher incidence of sepsis in patients with higher AAR. Second, elevated AAR levels could indicate increased glycolysis. A meta-analysis links high preoperative AAR in urothelial carcinoma patients to poor outcomes, suggesting heightened glycolysis in tumor cells [21]. Critical illness often induces a hypermetabolic state, and when mitochondrial function is impaired, cells may compensate for the deficiency in ATP production by enhancing glycolysis, which may subsequently lead to metabolic imbalances and other adverse conditions. Third, elevated AAR levels could point toward frailty and sarcopenia. Research has demonstrated a significant relationship between elevated AAR and sarcopenia, which is common in ICU patients and associated with higher mortality in critical illness [7,22]. However, the precise mechanisms linking AAR to mortality in ICU patients require further investigation.

This research is strengthened by several advantages, including its expansive sample size, extended follow-up duration, adjustments for a variety of significant risk factors and possible confounding factors, and a thorough examination of AAR’s association with mortality, as well as its impact across various subgroups. Furmore, the use of an external validation cohort from the eICU-CRD enhanced the generalizability and robustness of the study. Nonetheless, there are limitations to consider. Firstly, its retrospective approach is vulnerable to selection and ascertainment bias, despite the use of a multivariable Cox regression model to ensure analytical rigor. Secondly, the lack of detailed cause-of-death information in the MIMIC-IV database hinders a more nuanced competing risks analysis. Thirdly, the effectiveness of interventions aimed at modifying AST/ALT levels for better patient outcomes remains unclear. Hence, our results require confirmation from well-conducted, multi-center, and prospective studies.

## Conclusion

Our study has confirmed AAR as a crucial indicator for predicting the 28-day mortality among ICU patients, with this predictive ability extending across diverse patient subgroups. Furthermore, an inverted L-shaped correlation was identified between AAR and the risk of 28-day, 60-day, 90-day, and 365-day mortality for ICU patients. This finding suggests that AAR may serve as a robust biomarker for patient risk stratification and could inform clinical decision making within the ICU setting.

## Supporting information

S1 Table Univariate regression analysis of covariates and 28-day mortality in the MIMIC-IV database. (DOCX)

S2 Table Sensitivity analysis of AAR and 28-day mortality in the MIMIC-IV database. (DOCX)

S3 Table Baseline characteristics of the included patients in the eICU-CRD. (DOCX)

S4 Table Cox model analysis of AAR and In-hospital mortality in the eICU-CRD. (DOCX)

S1 Fig Flowchart of the patient selection in the eICU-CRD. (TIF)

S2 Fig RCS regression analysis of AAR with 60-day (a), 90-day (b) and 365-day (c) mortality in the MIMIC-IV database. Only 99.9% of the data is shown. The median AAR was defined as the reference standard. The pink area represents the 95% CI. Adjusted for all factors in Model 4.
(TIF)

S3 Fig RCS regression analysis of AAR and in-hospital mortality in the eICU-CRD. Only 99.9% of the data is shown. The median AAR was defined as the reference standard. The pink area represents the 95% CI. Adjusted for all factors in Model 4.
(TIF)

## Abbreviations

AAR: aspartate aminotransferase to alanine aminotransferase ratio
AST: aspartate aminotransferase
ALT: alanine aminotransferase
Q: quartile
RCS: restricted cubic splines
MIMIC-IV: Medical Information Mart for Intensive Care IV
HR: hazard ratios
CI: confidence intervals
bpm: beats per minute
SBP: systolic blood pressure
DBP: diastolic blood pressure
WBC: white blood cell
BUN: blood urea nitrogen
PT: prothrombin time
SOFA: sequential organ failure assessment
OASIS: Oxford acute severity of illness score
IQR: interquartile range
VIF: variance inflation factor
ICU: intensive care unit
COVID-19: coronavirus disease 2019
STROBE: Strengthening the Reporting of Observational studies in Epidemiology
ICD: International Classification of Diseases
