# Supplementary material for: Association between aspartate aminotransferase to alanine aminotransferase ratio and 28-day mortality of ICU patients: A retrospective cohort study from MIMIC-IV database
Source: PLoS One. 2025 May 23;20(5):e0324904. doi: 10.1371/journal.pone.0324904 (PMC12101646; doi:10.1371/journal.pone.0324904)
Supplement: S1 Table — (DOCX) [file pone.0324904.s001.docx]

**S1 Table.** Univariate regression analysis of covariates and 28-day mortality.

| **Variables** | **HR(95%CI)** | **P value** |
| --- | --- | --- |
| Male vs Female | 0.97 (0.91,1.03) | 0.328 |
| Age | 1.02 (1.02,1.03) | < 0.001 |
| Weight | 0.9951 (0.9937,0.9965) | < 0.001 |
| Others vs Married | 1.09 (1.02,1.16) | 0.012 |
| White vs Non-white | 0.8 (0.75,0.85) | < 0.001 |
| Smoking | 0.82 (0.75,0.9) | < 0.001 |
| Heart rate | 1.02 (1.01,1.02) | < 0.001 |
| SBP | 0.97 (0.97,0.98) | < 0.001 |
| DBP | 0.97 (0.96,0.97) | < 0.001 |
| Respiratory rate | 1.1 (1.09,1.11) | < 0.001 |
| SpO2, % | 0.96 (0.96,0.96) | < 0.001 |
| Glucose | 1.00(1.00,1.00) | 0.901 |
| Hemoglobin | 0.89 (0.88,0.9) | < 0.001 |
| Platelets | 0.9981 (0.9977,0.9984) | < 0.001 |
| WBC | 1.0063 (1.0055,1.0072) | < 0.001 |
| Anion gap | 1.07 (1.07,1.08) | < 0.001 |
| BUN | 1.01 (1.01,1.01) | < 0.001 |
| Creatinine | 1.09 (1.08,1.1) | < 0.001 |
| Calcium | 1.06 (1.03,1.1) | < 0.001 |
| Potassium | 1.32 (1.28,1.35) | < 0.001 |
| Chloride | 1.0009 (0.996,1.0058) | 0.725 |
| Sodium | 1.02 (1.02,1.03) | < 0.001 |
| PT | 1.02 (1.02,1.02) | < 0.001 |
| ALT | 1 (1,1.0001) | < 0.001 |
| AST | 1.0001 (1.0001,1.0001) | < 0.001 |
| Myocardial infarct | 1.35 (1.25,1.46) | < 0.001 |
| Congestive heart failure | 1.31 (1.23,1.4) | < 0.001 |
| Coronary heart disease | 1.09 (0.99,1.2) | 0.089 |
| Atrial fibrillation | 1.44 (1.34,1.55) | < 0.001 |
| Peripheral vascular disease | 1.23 (1.12,1.36) | < 0.001 |
| Cerebrovascular disease | 1.46 (1.35,1.59) | < 0.001 |
| Dementia | 1.82 (1.6,2.07) | < 0.001 |
| Chronic pulmonary disease | 1.13 (1.05,1.21) | 0.001 |
| Rheumatic disease | 1.11 (0.93,1.31) | 0.247 |
| Liver disease | 1.6 (1.49,1.72) | < 0.001 |
| Renal disease | 1.4 (1.3,1.51) | < 0.001 |
| Hypertension | 1.11 (1.04,1.19) | 0.001 |
| Diabetes | 1.03 (0.96,1.1) | 0.448 |
| Sepsis | 2.29 (2.13,2.46) | < 0.001 |
| Charlson comorbidity index | 1.18 (1.16,1.19) | < 0.001 |
| OASIS | 1.09 (1.08,1.09) | < 0.001 |
| SOFA | 1.21 (1.2,1.22) | < 0.001 |
| ASL/ALT ratio | 1.1 (1.09,1.1) | < 0.001 |
| ASL/ALT ratio group |  |  |
| Q1 ＜1.035 | 1(Ref) |  |
| Q2 (1.053-1.441) | 1.26 (1.13,1.4) | < 0.001 |
| Q3 (1.441-2.055) | 1.81 (1.64,2) | < 0.001 |
| Q4 ≥2.056 | 2.46 (2.24,2.7) | < 0.001 |
