# Supplementary material for: Association between aspartate aminotransferase to alanine aminotransferase ratio and 28-day mortality of ICU patients: A retrospective cohort study from MIMIC-IV database
Source: PLoS One. 2025 May 23;20(5):e0324904. doi: 10.1371/journal.pone.0324904 (PMC12101646; doi:10.1371/journal.pone.0324904)
Supplement: S2 Table — (DOCX) [file pone.0324904.s002.docx]

**S2 Table.** Sensitivity analysis of AAR and 28-day mortality.

| **Variable** | **Model** 1 | | **Model** 2 | | **Model** 3 | | **Model** 4 | |
| --- | --- | --- | --- | --- | --- | --- | --- | --- |
|  | **HR(95%)** | **P value** | **HR(95%)** | **P value** | **HR(95%)** | **P value** | **HR(95%)** | **P value** |
| AAR | 1.09 (1.08~1.1) | <0.001 | 1.1 (1.09~1.11) | <0.001 | 1.07 (1.06~1.09) | <0.001 | 1.05 (1.03~1.06) | <0.001 |
| AAR quartiles |  |  |  |  |  |  |  |  |
| Q1＜1.035 | 1 (Reference) |  | 1 (Reference) |  | 1 (Reference) |  | 1 (Reference) |  |
| Q2 (1.035-1.441) | 1.27 (1.14~1.42) | <0.001 | 1.18 (1.05~1.32) | 0.004 | 1.15 (1.03~1.29) | 0.014 | 1.09 (0.98~1.22) | 0.125 |
| Q3 (1.441-2.055) | 1.9 (1.71~2.11) | <0.001 | 1.71 (1.54~1.9) | <0.001 | 1.49 (1.34~1.65) | <0.001 | 1.34 (1.2~1.49) | <0.001 |
| Q4 ≥2.056 | 2.59 (2.35~2.87) | <0.001 | 2.42 (2.19~2.68) | <0.001 | 1.8 (1.62~2) | <0.001 | 1.47 (1.33~1.64) | <0.001 |
| P for trend |  | <0.001 |  | <0.001 |  | <0.001 |  | <0.001 |

Model 1: no covariates were adjusted.

Model 2: adjusted for age, gender, weight, race, smoking, and marital status.

Model 3: adjusted for mode 2 + heart rate, SBP, DBP, Respiratory rate, SpO2, hemoglobin, platelets, WBC, aniongap, bun, creatinine, potassium, sodium, and PT.

Model 4: adjusted for mode 3 + myocardial infarct, congestive heart failure, atrial fibrillation, peripheral vascular disease, cerebrovascular disease, dementia, chronic pulmonary disease, liver disease, renal disease, hypertension, diabetes, sepsis, Charlson comorbidity index, OASIS, and SOFA.
