# Supplementary material for: Association between aspartate aminotransferase to alanine aminotransferase ratio and 28-day mortality of ICU patients: A retrospective cohort study from MIMIC-IV database
Source: PLoS One. 2025 May 23;20(5):e0324904. doi: 10.1371/journal.pone.0324904 (PMC12101646; doi:10.1371/journal.pone.0324904)
Supplement: S3 Table — (DOCX) [file pone.0324904.s003.docx]

**S3 Table.** Baseline characteristics of the included patients in eICU-CRD.

| **Characteristics** | **Total (n = 61105)** | **AAR quartiles** | | | | **P value** |
| --- | --- | --- | --- | --- | --- | --- |
|  |  | **Q1 < 0.875 (n = 15275)** | **Q2 (0.875-1.244) (n = 15277)** | **Q3 (1.244-1.809)  (n = 15258)** | **Q4 ≥ 1.809 (n = 15295)** |  |
| **Demographics** | | | | | | |
| Age, years | 62.2 ± 17.1 | 59.1 ± 17.4 | 62.6 ± 17.2 | 64.1 ± 17.0 | 63.1 ± 16.3 | < 0.001 |
| Male, n (%) | 33226 (54.4) | 8841 (57.9) | 8251 (54) | 7884 (51.7) | 8250 (53.9) | < 0.001 |
| Race, n (%) |  |  |  |  |  | < 0.001 |
| Non-white | 14607 (23.9) | 3514 (23) | 3473 (22.7) | 3714 (24.3) | 3906 (25.5) |  |
| White | 46498 (76.1) | 11761 (77) | 11804 (77.3) | 11544 (75.7) | 11389 (74.5) |  |
| Weight, Kg | 83.7 ± 26.8 | 87.2 ± 28.0 | 84.3 ± 26.3 | 82.0 ± 27.0 | 81.5 ± 25.7 | < 0.001 |
| **Vital signs** |  |  |  |  |  |  |
| Heart rate, bpm | 103.2 ± 31.4 | 101.2 ± 31.2 | 101.5 ± 31.2 | 103.5 ± 31.5 | 106.5 ± 31.3 | < 0.001 |
| Respiratory rate, bpm | 28.0 (11.0, 36.0) | 27.0 (11.0, 35.0) | 28.0 (11.0, 36.0) | 29.0 (11.0, 37.0) | 29.0 (11.0, 37.0) | < 0.001 |
| MeanBP, mmHg | 65.0 (52.0, 124.0) | 68.0 (55.0, 124.0) | 66.0 (53.0, 126.0) | 64.0 (52.0, 125.0) | 61.0 (50.0, 123.0) | < 0.001 |
| **Laboratory data** | | | | | | |
| Hemoglobin, g/dL | 11.1 ± 2.4 | 11.5 ± 2.4 | 11.3 ± 2.3 | 10.9 ± 2.3 | 10.8 ± 2.4 | < 0.001 |
| Anion gap, mmol/L | 11.5 ± 5.0 | 10.8 ± 4.6 | 11.0 ± 4.6 | 11.6 ± 4.9 | 12.5 ± 5.6 | < 0.001 |
| Potassium, mmol/L | 4.1 ± 0.7 | 4.1 ± 0.7 | 4.1 ± 0.7 | 4.1 ± 0.7 | 4.1 ± 0.8 | < 0.001 |
| Sodium, mmol/L | 126.1 ± 39.1 | 125.0 ± 41.1 | 125.8 ± 39.9 | 126.6 ± 38.3 | 127.0 ± 37.1 | < 0.001 |
| Platelets, K/µL | 191.0 (140.0, 251.0) | 206.0 (158.0, 263.0) | 196.0 (149.0, 253.0) | 188.0 (137.0, 247.0) | 172.0 (115.0, 235.0) | < 0.001 |
| WBC, K/µL | 9.3 (5.8, 14.8) | 8.9 (5.8, 13.5) | 9.1 (5.8, 14.2) | 9.4 (5.8, 15.1) | 10.1 (5.9, 16.3) | < 0.001 |
| BUN, mg/dL | 19.0 (11.0, 34.0) | 17.0 (11.0, 30.0) | 18.0 (11.0, 32.0) | 20.0 (12.0, 35.0) | 21.0 (12.0, 38.0) | < 0.001 |
| Creatinine, mg/dL | 0.9 (0.7, 1.7) | 0.9 (0.6, 1.4) | 0.9 (0.6, 1.5) | 1.0 (0.7, 1.8) | 1.1 (0.7, 2.1) | < 0.001 |
| ALT, IU/L | 26.0 (16.0, 49.0) | 34.0 (23.0, 60.0) | 25.0 (17.0, 43.0) | 21.0 (14.0, 41.0) | 25.0 (13.0, 51.0) | < 0.001 |
| AST, IU/L | 31.0 (19.0, 67.0) | 22.0 (15.0, 37.0) | 26.0 (18.0, 45.0) | 32.0 (20.0, 62.0) | 65.0 (33.0, 154.0) | < 0.001 |
| AST/ALT ratio | 1.2 (0.9, 1.8) | 0.7 (0.6, 0.8) | 1.1 (1.0, 1.1) | 1.5 (1.4, 1.6) | 2.5 (2.1, 3.2) | < 0.001 |
| **Comorbidities, n (%)** | | | | | | |
| Myocardial infarct | 5273 ( 8.6) | 1195 (7.8) | 1339 (8.8) | 1330 (8.7) | 1409 (9.2) | < 0.001 |
| Congestive heart failure | 9091 (14.9) | 2079 (13.6) | 2337 (15.3) | 2455 (16.1) | 2220 (14.5) | < 0.001 |
| Peripheral vascular disease | 2615 ( 4.3) | 517 (3.4) | 634 (4.2) | 700 (4.6) | 764 (5) | < 0.001 |
| Cerebrovascular disease | 6048 ( 9.9) | 1426 (9.3) | 1592 (10.4) | 1604 (10.5) | 1426 (9.3) | < 0.001 |
| Dementia | 1980 ( 3.2) | 381 (2.5) | 488 (3.2) | 584 (3.8) | 527 (3.4) | < 0.001 |
| Chronic pulmonary disease | 8987 (14.7) | 2344 (15.3) | 2299 (15) | 2325 (15.2) | 2019 (13.2) | < 0.001 |
| Renal disease | 8004 (13.1) | 1702 (11.1) | 1936 (12.7) | 2154 (14.1) | 2212 (14.5) | < 0.001 |
| Liver disease | 2774 ( 4.5) | 250 (1.6) | 418 (2.7) | 734 (4.8) | 1372 (9) | < 0.001 |
| Diabetes | 18303 (30.0) | 4917 (32.2) | 4748 (31.1) | 4505 (29.5) | 4133 (27) | < 0.001 |
| Hypertension | 7816 (12.8) | 2236 (14.6) | 2045 (13.4) | 1911 (12.5) | 1624 (10.6) | < 0.001 |
| Atrial fibrillation | 5391 ( 8.8) | 1282 (8.4) | 1429 (9.4) | 1442 (9.5) | 1238 (8.1) | < 0.001 |
| Sepsis | 11140 (18.2) | 2416 (15.8) | 2552 (16.7) | 2922 (19.2) | 3250 (21.2) | < 0.001 |
| **Severity of illness** | | | | | | |
| Charlson comorbidity index | 3.0 (2.0, 5.0) | 3.0 (1.0, 5.0) | 4.0 (2.0, 5.0) | 4.0 (2.0, 6.0) | 4.0 (2.0, 6.0) | < 0.001 |
| OASIS | 28.2 ± 10.2 | 25.7 ± 9.2 | 27.5 ± 9.7 | 29.1 ± 10.3 | 30.8 ± 10.8 | < 0.001 |
| SOFA score | 5.0 (3.0, 8.0) | 4.0 (2.0, 6.0) | 5.0 (2.0, 7.0) | 5.0 (3.0, 8.0) | 6.0 (4.0, 9.0) | < 0.001 |
| Apache score | 60.8 ± 28.6 | 52.2 ± 24.0 | 57.8 ± 26.4 | 63.2 ± 28.3 | 69.6 ± 31.8 | < 0.001 |
| In-hospital mortality, n (%) | 6937 (11.4) | 852 (5.6) | 1298 (8.5) | 1853 (12.1) | 2934 (19.2) | < 0.001 |
| LOS in hospital, days | 5.8 (3.1, 10.2) | 5.0 (2.8, 9.1) | 5.5 (3.0, 9.8) | 6.1 (3.4, 10.6) | 6.4 (3.3, 11.5) | < 0.001 |
| LOS in ICU, days | 1.9 (1.0, 3.7) | 1.7 (0.9, 3.1) | 1.8 (1.0, 3.5) | 2.0 (1.1, 3.9) | 2.2 (1.2, 4.3) | < 0.001 |
