# Supplementary material for: Association between aspartate aminotransferase to alanine aminotransferase ratio and 28-day mortality of ICU patients: A retrospective cohort study from MIMIC-IV database
Source: PLoS One. 2025 May 23;20(5):e0324904. doi: 10.1371/journal.pone.0324904 (PMC12101646; doi:10.1371/journal.pone.0324904)
Supplement: S4 Table — (DOCX) [file pone.0324904.s004.docx]

**S4 Table.** Cox model analysis of AAR and In-hospital mortality in the eICU-CRD.

| **Variable** | **Model 1** | | **Model 2** | | **Model 3** | | **Model 4** | |
| --- | --- | --- | --- | --- | --- | --- | --- | --- |
|  | **HR(95%)** | **P value** | **HR(95%)** | **P value** | **HR(95%)** | **P value** | **HR(95%)** | **P value** |
| AAR | 1.15 (1.14~1.16) | <0.001 | 1.14 (1.14~1.15) | <0.001 | 1.1 (1.09~1.11) | <0.001 | 1.04 (1.03~1.05) | <0.001 |
| AAR quartiles |  |  |  |  |  |  |  |  |
| Q1 < 0.875 | 1 (Reference) |  | 1 (Reference) |  | 1 (Reference) |  | 1 (Reference) |  |
| Q2 (0.875-1.244) | 1.46 (1.34~1.59) | <0.001 | 1.39 (1.27~1.51) | <0.001 | 1.34 (1.22~1.46) | <0.001 | 1.16 (1.06~1.27) | 0.001 |
| Q3 (1.244-1.809) | 1.93 (1.78~2.1) | <0.001 | 1.82 (1.68~1.97) | <0.001 | 1.61 (1.48~1.74) | <0.001 | 1.28 (1.18~1.39) | <0.001 |
| Q4 ≥ 1.809 | 2.88 (2.67~3.11) | <0.001 | 2.8 (2.59~3.02) | <0.001 | 2.19 (2.03~2.37) | <0.001 | 1.51 (1.39~1.64) | <0.001 |

Model 1: no covariates were adjusted.

Model 2: adjusted for age, gender, weight, race.

Model 3: adjusted for mode 2 + heart rate, MeanBP, Respiratory rate, hemoglobin, platelets, WBC, aniongap, bun, creatinine, potassium, and, sodium.

Model 4: adjusted for mode 3 + myocardial infarct, congestive heart failure, atrial fibrillation, peripheral vascular disease, cerebrovascular disease, dementia, chronic pulmonary disease, liver disease, renal disease, hypertension, diabetes, sepsis, Charlson comorbidity index, OASIS, and, SOFA.
