# Supplementary figures and images for: Association between aspartate aminotransferase to alanine aminotransferase ratio and 28-day mortality of ICU patients: A retrospective cohort study from MIMIC-IV database
Source: PLoS One. 2025 May 23;20(5):e0324904. doi: 10.1371/journal.pone.0324904 (PMC12101646; doi:10.1371/journal.pone.0324904)

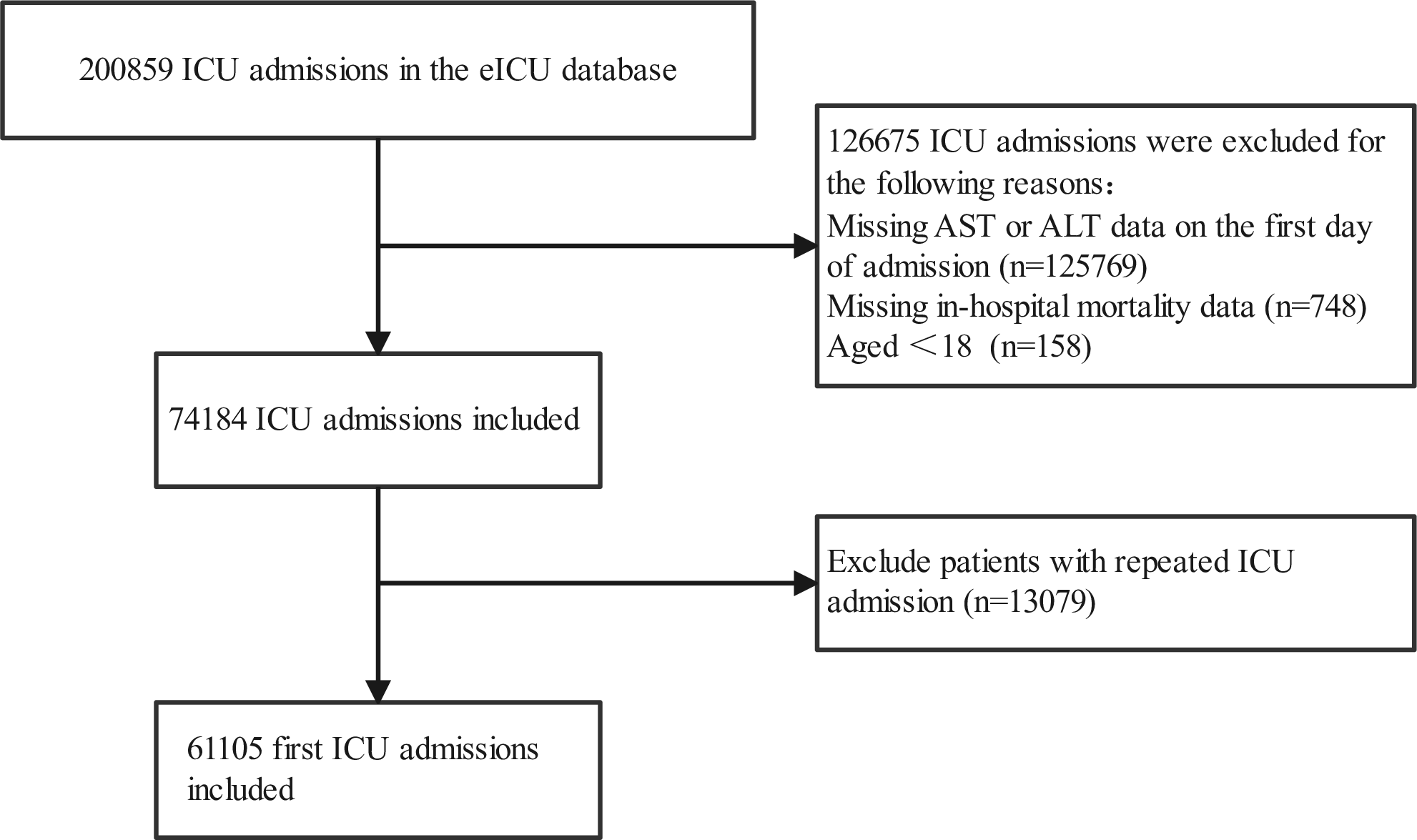

Supplement: S1 Fig — (TIF) [file pone.0324904.s005.tif]

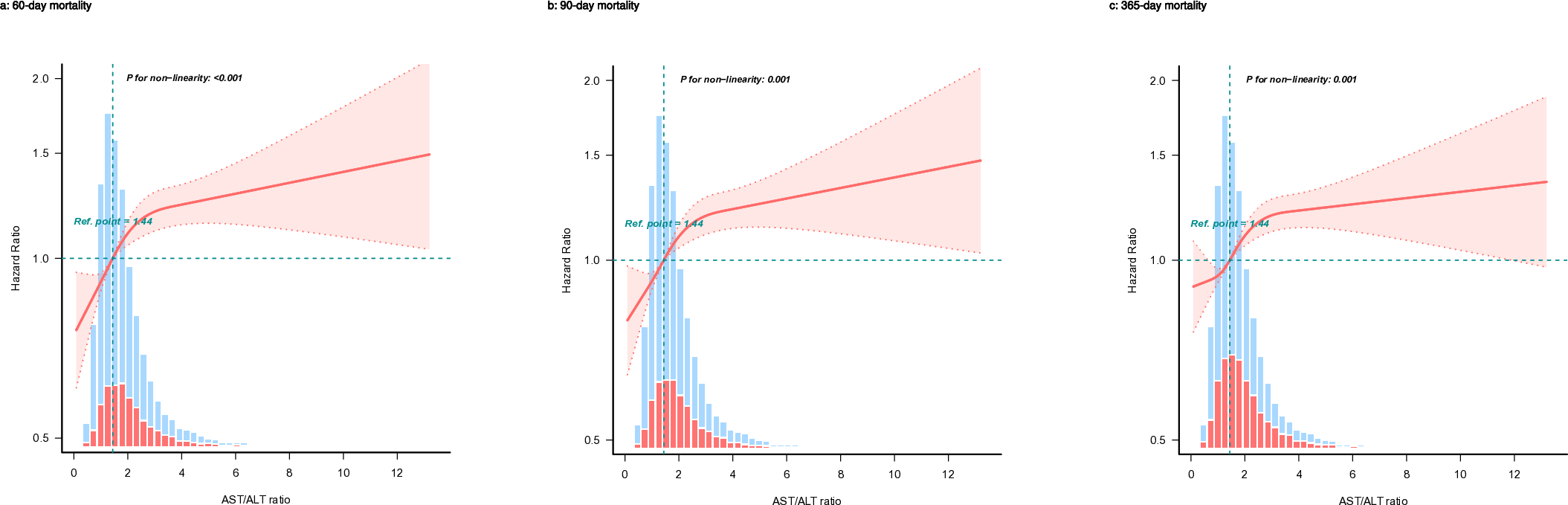

Supplement: S2 Fig — Only 99.9% of the data is shown. The median AAR was defined as the reference standard. The pink area represents the 95% CI. Adjusted for all factors in Model 4. (TIF) [file pone.0324904.s006.tif]

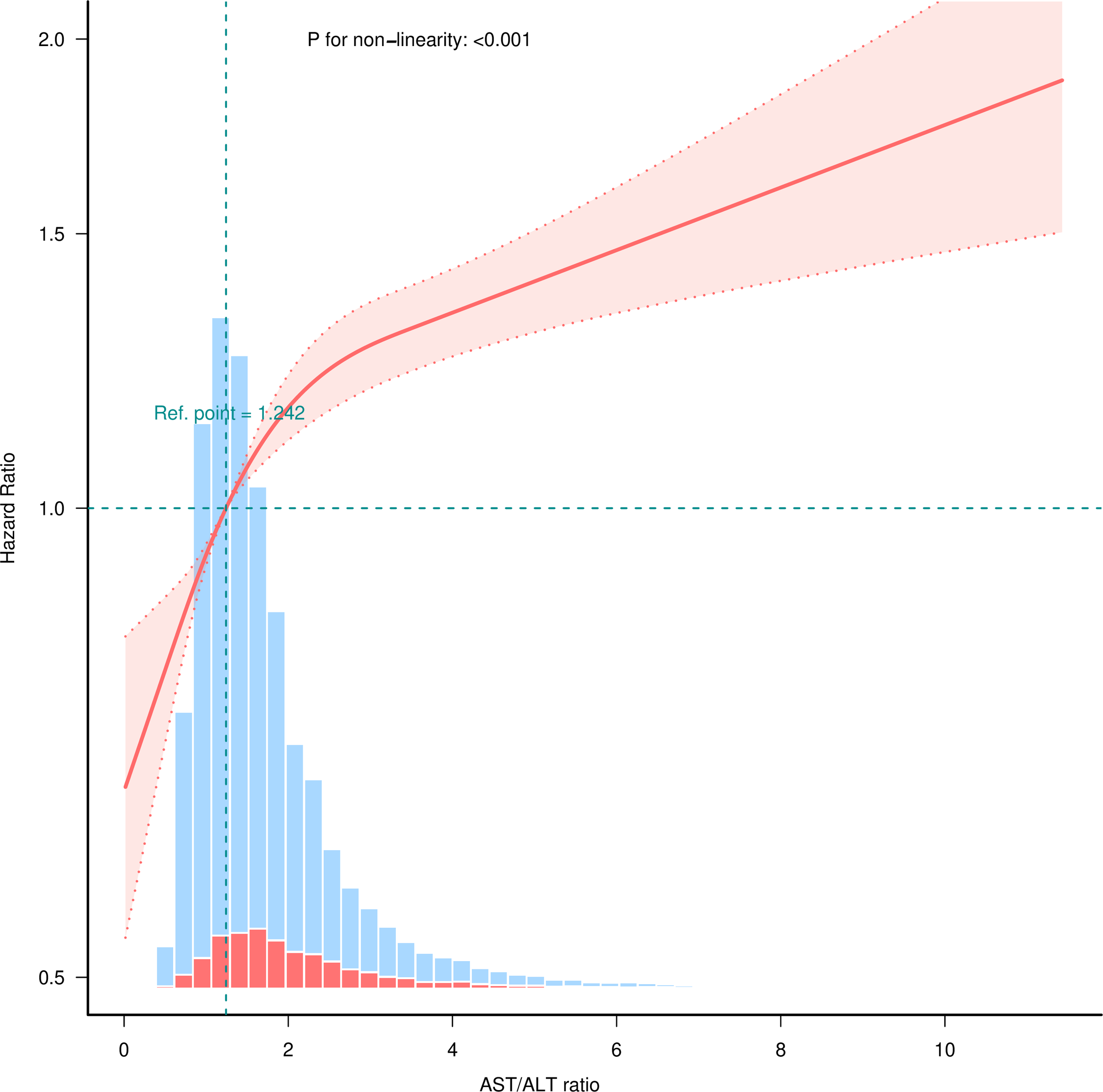

Supplement: S3 Fig — Only 99.9% of the data is shown. The median AAR was defined as the reference standard. The pink area represents the 95% CI. Adjusted for all factors in Model 4. (TIF) [file pone.0324904.s007.tif]
